# Ophthalmological and electrophysiological findings in monozygotic twin sisters with phosphomannomutase 2 deficiency (PMM2-CDG) over a period of 37 years

**DOI:** 10.3205/oc000126

**Published:** 2019-11-20

**Authors:** Ines Van Hees, Jaak Jaeken, Wouter Meersseman, Ingele Casteels

**Affiliations:** 1 Department of Ophthalmology, University Hospitals Leuven, Belgium; 2 Department of Paediatrics and Center for Metabolic Diseases, University Hospitals Leuven, Belgium; 3 Department of General Internal Medicine and Center for Metabolic Diseases, University Hospitals Leuven, Belgium

## Abstract

**Aims:** To describe the evolution of ophthalmological and electrophysiological findings in monozygotic twin sisters with phosphomannomutase 2 deficiency (PMM2-CDG).

**Methods:** A clinical ophthalmological examination with visual acuity measurement, fundoscopy and flash electroretinogram (fERG) was performed at the age of 4, 18 and 41 years.

**Results:** Ophthalmic examination in both girls at the age of 4 years showed an alternating convergent squint and a saccadic pursuit, with visual acuity of 6/9 in both eyes (Ffooks symbols test). Fundoscopy revealed a normal aspect of the optic discs, narrowed blood vessels and a mild irregular pigmentation in the peripheral retina. Flash ERG in one girl showed a recognisable a, b1 and b2 wave, but with a reduction of the amplitude to less than 40% of the normal amplitude. In the other twin girl, the amplitude was more reduced, but a small b1 wave for the white flash was still noticeable. At the age of 18 years, vision had remained stable. Fundus examination revealed a pink aspect of the optic discs, with moderately narrowing of the vasculature and bone spicules in the mid peripheral retina. fERG showed obvious progression with a completely extinguished trace bilaterally. At the age of 41 years, vision had slightly diminished to 6/12 in both women. Fundoscopy and electroretinogram did not show any changes.

**Conclusions:** Despite obvious deterioration of the fERG between the age of 4 and 18 years, the central vision showed only a minor decrease between the age of 18 and 41 years with still a good functional visual acuity.

## Introduction

Congenital disorders of glycosylation (CDG) encompass a group of genetic, mostly multisystem disorders with involvement of the nervous system and the eyes caused by a defective glycoprotein and glycolipid glycan synthesis and attachment. The large majority has an autosomal recessive inheritance. Some 130 different CDG cases have been reported since the first clinical description of phosphomannomutase deficiency (PMM2-CDG) in 1980 by Jaak Jaeken [1], [2], [3]. PMM2-CDG is by far the most frequent protein N-glycosylation disorder. A recent review on ophthalmological findings in protein N-glycosylation disorders has been published by Morava et al. [4], and on protein O-glycosylation disorders by Francisco et al. [5]. Characteristic ophthalmic findings of PMM2-CDG are convergent strabismus and retinitis pigmentosa with abnormal electroretinography and visual evoked potential findings [3], [4], [6], [7], [8], [9], [10], [11], [12], [13].

We report the ophthalmic findings and evolution over a period of 37 years – both ocular and electroretinographic – in monozygotic twin sisters with PMM2-CDG, in follow-up of a 1996 report by Casteels et al. [14].

## Case description

**Figure 1:**
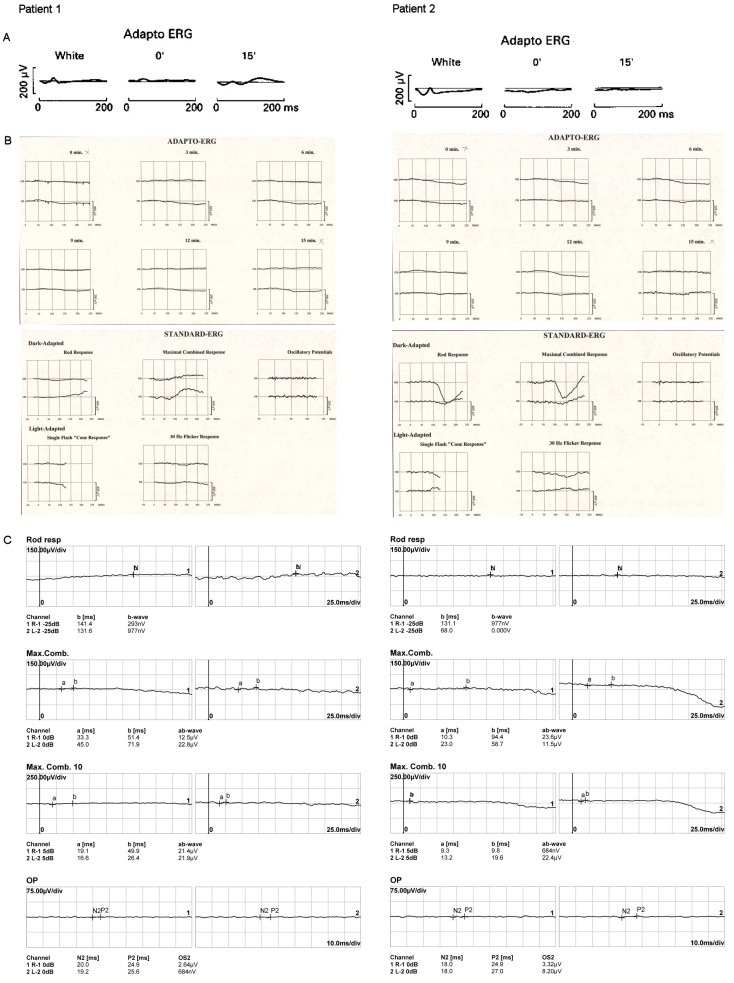
Electroretinographic findings in twin sisters (patient 1 and patient 2) with PMM2-CDG at the age of 4 years (A), 18 years (B) and 41 years (C). A: 1980: Adapto ERG revealed decreased amplitudes, more obvious for patient 2. B: 1995: On adapto ERG, repsonses were entirely extinguished bilaterally. This exam was now completed with an ERG using the ISCEV standard; no recordable dark adapted or light adapted response was observed. C: 2018: ERG revealed unchanged findings.

The princeps patients (monozygotic twin girls) with an intermediate form of PMM2-CDG (compound heterozygous with mutations C.338C>T (p.P113L) and C.422G>A (p.R141H)) presented to the ophthalmic department at the age of 4 years [15]. Clinical examination of the eye movements showed an alternating convergent squint and a saccadic pursuit. Vision in both eyes was 6/9 (logMAR 0.22) measured with the Ffooks symbols test; there was no refractive error on retinoscopy. In both subjects, fundoscopy revealed a normal aspect of the optic discs, with a narrowing of the blood vessels and mild irregular pigmentation in the peripheral retina [14]. After pupillary dilatation with 15% phenylephrine and 1% cyclopentolate hydrochloride, a full field flash electroretinogram (fERG) was obtained using bipolar contact lens electrodes and a ground electrode on the forehead. During a period of light adaptation to suppress the effect of rods, the isolated cone response can be measured. A white flash is shown immediately followed by a dim orange flash (using a Wratten orange number 26 filter) and the ERG responses to these stimuli are measured (respectively white and 0’ in Figure 1A (Fig. 1)). A normal cone response consists of an a-wave and a b1-wave. These responses are followed by a period of 15’ of dark adaptation to enlarge rod contribution of the ERG response to the orange flash (15’ in Figure 1A (Fig. 1)). In normal subjects, a later b2 rod wave will follow the initial a-wave and b1-wave. For patient 1 (Figure 1A (Fig. 1)), these 3 waves could be clearly seen, but their amplitudes were decreased to less than 40% of the normal amplitude. This decrease in amplitude was even more obvious in patient 2 (Figure 1A (Fig. 1)). A small b1-wave could still be seen as a response to the white flash, however the orange flash did not give rise to any recognisable response [14], [16].

**Figure 2:**
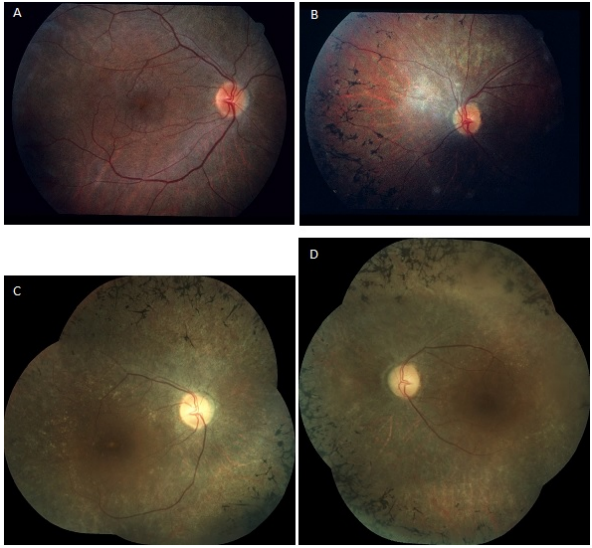
Fundus appearance in twin sisters with PMM2-CDG at the age of 18 and 41 years. (A,B) At the age of 18 years, a pink aspect of the optic discs and a moderate narrowing of the vasculature could be seen in addition to bony spicule pigmentary deposits in the mid peripheral retina, both in patient 1 (A) and patient 2 (B). In patient 1, a wrinkling of the macular retinal surface was noticed (A). (C,D) At the age of 41 years, findings of fundoscopy had remained the same for patient 1 (C) and patient 2 (D).

An ophthalmic reevaluation was performed at the age of 18. Both girls were wheelchair bound and mentally disabled. Their parents did not notice obvious visual loss or night blindness. Vision had remained stable at 6/9 (logMAR 0.22) for distance and near in both girls, measured with the Ffooks symbols test. Saccadic pursuit was still obvious. Ishihara colour vision testing revealed no abnormalities. Confrontation test showed constriction of the peripheral visual fields. On examination of the fundus, a pink aspect of the optic discs and a moderately narrowing of the blood vessels could be seen in addition to bony spicule pigmentary deposits in the mid peripheral retina. The latter was more obvious in the first patient. In the other patient a wrinkling of the macular retinal surface was noticed (Figure 2A,B (Fig. 2)). Adapto ERG was achieved in equal conditions as 14 years earlier. At the age of 18 years, responses were completely extinguished bilaterally. This adapto ERG exam was now completed with an ERG using the ISCEV standard (Figure 1B (Fig. 1)). No recordable dark adapted or light adapted response was observed [14], [17].

**Figure 3:**
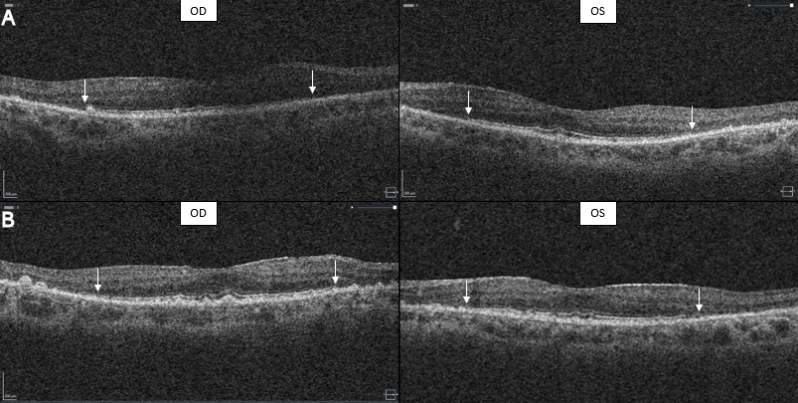
OCT examination in twin sisters with PPM2-CDG at the age of 41 For patient 1 (A) and patient 2 (B), severe attenuation of the outer retina starting from the perimacular area is shown. In the central macular area, the normal outer retinal structure was preserved. Drusenoïd like changes can also be observed, more obvious in the second patient.

Both girls were reassessed at the age of 41. Their parents did not note any change in visual performance. The internal strabismus had become less obvious over the years. Examination of the vision with the Ffooks symbols test showed a slight decrease to 6/12 (logMAR 0.3) for distance and 6/24 (logMAR 0.6) for near. Saccadic pursuit was still present. Ishihara colour vision testing remained normal. Testing of the peripheral visual fields by Goldmann perimetry and confrontation test revealed concentric narrowing. Findings of fundoscopy remained unchanged: pink optic discs, moderately narrowed blood vessels and bony spicule pigmentary deposits in the mid periphery (Figure 2C,D (Fig. 2)). The ERG examination showed completely extinguished traces for both eyes, as at the age of 18 (Figure 1C (Fig. 1)). Optical coherence tomography (OCT) showed severe attenuation of the outer retina starting from the perimacular area; in the central macular area, the normal outer retinal structure was preserved. Drusenoïd-like changes could also be observed, more obvious in the second patient (Figure 3A,B (Fig. 3)) [17].

## Discussion

Characteristic ophthalmic findings in PMM2-CDG include convergent squint and retinal dystrophy with abnormalities on electroretinography. The majority of patients also show visual field loss with consequently progressive loss of vision. Other reported ocular manifestations include progressive myopia, hyperopia, cataract, nyctalopia, delayed visual maturation and abnormal eye movements [4], [6], [18], [19]. The importance of CDG as a metabolic cause of retinal dystrophy with bony spicule pigmentary deposits has been reported by Fiumara et al. According to these authors, CDG should be considered in cases with early onset of strabismus followed by unexplained retinopathy. Isoelectrofocusing of serum sialotransferrins, the standard screening test, should be performed in these patients [6]. Andreasson et al. studied the electrophysiological findings in five patients with a PMM2-CDG phenotype. Only two of them showed the typical findings on fundoscopy of retinitis pigmentosa, whereas the electroretinogram was abnormal in all five patients. Their observations were suggestive of a progressive tapetoretinal disorder with defined alterations in the ERG [20].

Our patients presented at the pediatric department with an alternating convergent squint at the age of 19 months. Orthoptic examination showed an impaired abduction in both eyes. On attempted lateral gaze, a jerk nystagmus was noted. Testing of eye motility revealed no smooth pursuit but saccadic eye movements. In a clinical overview, Jaeken et al. described the same ophthalmic finding in all 29 children during their first year of life [9], [14]. Examination of the fundus at the age of 4 years showed typical signs of retinitis pigmentosa with narrowed vessels and mild pigmentary deposits. Findings on fERG were also very suggestive of a retinal dystrophy. Central vision was 6/9 (logMAR 0.22) for distance and near for both girls at that age and had remained unchanged 14 years later. At the age of 18, constricted peripheral fields were obvious on confrontation test. Fundoscopy revealed progression of the pigmentary deposits with mid peripheral bony spicules. The electroretinogram showed a total absence of rod and cone function. At the age of 41, central vision had slightly diminished to 6/12 (logMAR 0.3) for distance and 6/24 (logMAR 0.6) for near. Findings of fundoscopy, peripheral field testing and fERG had remained the same [14]. OCT – for the first time performed at that age – revealed severe attenuation of the outer retina starting from the perimacular area with preservation of the normal outer retinal structure in the central macular area.

In our patients, both funduscopic and electroretinographic findings showed obvious progression of retinitis pigmentosa during the first follow-up period of 14 years, with an extinguished ERG response for rods and cones. These findings are consistent with those of Fiumara et al. in their description of the ophthalmic aspects in four patients with PMM2-CDG [6]. During the further follow-up period of 23 years, only mild deterioration of visual acuity but stable visual fields and ophthalmoscopic findings could be demonstrated. 

As strabismus is an early symptom in patients with PMM2-CDG, amblyopia could be a possible reason for the reduction of visual acuity. Though, this was not the case in our patients. Often the squint is alternating as was seen in both women. Because of its alternating aspect, the chance to develop amblyopia is much smaller. A majority of the children with PMM2-CDG develop visual field loss due to retinitis pigmentosa, eventually leading to progressive visual loss [4]. The main systemic symptom of both patients at any age is psychomotor disability. This has remained stable over the years and cannot explain a visual decline either.

The findings on OCT, namely preservation of normal outer retinal structure in the central macular area, are consistent with the study of Messenger et al. and could explain the maintenance of central vision in both patients [19]. In a study of Thompson et al., pattern VEP showed functional preservation of the macular pathways to the recipient layer 4 of the striate cortex. They propose that this finding could be the reason why most patients keep a functional vision [11]. These findings are congruent with previous reports that the retinal dysfunction spares the central part of the retina that is responsible for visual acuity [20].

In conclusion, this report is a follow-up report to the publication by Casteels et al. in 1996 describing the evolution of visual function and electrophysiological findings in twin sisters with PMM2-CDG over a period of 37 years [14]. Despite obvious deterioration of the retinal function on flash ERG between the age of 4 years and 18 years, the central vision showed only a mild deterioration between the age of 18 years and 41 years with continuing good functional visual acuity. To our knowledge, this report describes the longest ophthalmological follow-up of patients with PMM2-CDG.

## Notes

### Competing interests

The authors declare that they have no competing interests.
